# Role of docosahexaenoic acid in the modulation of glial cells in Alzheimer’s disease

**DOI:** 10.1186/s12974-016-0525-7

**Published:** 2016-03-10

**Authors:** David Heras-Sandoval, José Pedraza-Chaverri, Jazmin M. Pérez-Rojas

**Affiliations:** Departamento de Biología, Facultad de Química, Universidad Nacional Autónoma de México, Ciudad Universitaria, 04510 México, DF México; Laboratorio de Farmacología, Subdirección de Investigación Básica, Instituto Nacional de Cancerología (INCan), Av. San Fernando #22, Tlalpan 14080, Apartado Postal 22026 México, DF México

**Keywords:** Amyloid β peptide (Aβ), Alzheimer’s disease (AD), Docosahexaenoic acid (DHA), Neuroinflammation, Neuroprotectin D1 (NPD1), Resolvin D1 (RvD1), Glial cells

## Abstract

Docosahexaenoic acid (DHA) is an omega-3 (ω-3) long-chain polyunsaturated fatty acid (LCPUFA) relevant for brain function. It has largely been explored as a potential candidate to treat Alzheimer’s disease (AD). Clinical evidence favors a role for DHA in the improvement of cognition in very early stages of the AD. In response to stress or damage, DHA generates oxygenated derivatives called docosanoids that can activate the peroxisome proliferator-activated receptor γ (PPARγ). In conjunction with activated retinoid X receptors (RXR), PPARγ modulates inflammation, cell survival, and lipid metabolism. As an early event in AD, inflammation is associated with an excess of amyloid β peptide (Aβ) that contributes to neural insult. Glial cells are recognized to be actively involved during AD, and their dysfunction is associated with the early appearance of this pathology. These cells give support to neurons, remove amyloid β peptides from the brain, and modulate inflammation. Since DHA can modulate glial cell activity, the present work reviews the evidence about this modulation as well as the effect of docosanoids on neuroinflammation and in some AD models. The evidence supports PPARγ as a preferred target for gene modulation. The effective use of DHA and/or its derivatives in a subgroup of people at risk of developing AD is discussed.

## Background

Docosahexaenoic acid (DHA), an omega-3 (ω-3) long-chain polyunsaturated fatty acid (LCPUFA), is involved in a wide range of cellular processes in mammalian cells. It serves as a structural component and as a precursor for bioactive compounds that modulate cell signaling and gene expression [1]. Humans lack Δ15-desaturase and Δ12-desaturase, and they do not produce αLNA de novo, and it is from this compound that eicosapentaenoic acid (EPA) and ultimately DHA are produced [2, 3]. Consequently, humans need to acquire DHA or its precursors, α-linolenic acid (αLNA) and EPA, from food in order to fulfill bodily requirements [2, 4]. DHA and its precursors are present in oils from macroalgae [3] and cold water fish, from the latter in relatively large quantities [1]. Alternatively, DHA can be synthesized from its precursor αLNA, which is found in oils from seeds and green leafy vegetables [1, 3, 5].

### DHA absorption and synthesis from αLNA and EPA

During fetal life and breast feeding, DHA is obtained from the mother. After weaning, DHA derives from animal food, especially fish [1, 5, 6]. In nature, ω-3 fatty acids are mainly esterified as triacylglycerol (TAG) and phospholipids (PLs) [2, 7]. TAG is hydrolyzed in the digestive tract by lingual, pancreatic, and gastric lipases to yield monoacylglycerols and free LCPUFAs [2, 7]. PLs are hydrolyzed in the small intestine by calcium-independent phospholipase A_2_ (iPLA_2_) and other lipases [7]. The products of TAG and PL hydrolysis are then absorbed by enterocytes and reassembled into TAG and PLs, which are then integrated into chylomicrons, high-density lipoproteins, and very low-density lipoproteins, finally reaching the liver via the lymphatic system and the blood stream [2, 7].

Alternatively, DHA can be obtained from αLNA or EPA through chain elongation and desaturation processes in the liver, involving Δ6-desaturase (Δ6D), elongase complex activity, Δ5-desaturase, and β-oxidation of tetracosahexaenoic acid to form DHA in the peroxisomes [8, 9]. Although αLNA is considered a poor source for DHA production [4, 10, 11], a diet containing only αLNA can give adequate amounts of DHA in human and rat livers [12, 13]. Nevertheless, higher levels of αLNA and linoleic acid (LA) can potentially inhibit Δ6D and DHA production through β-oxidation [14]. After DHA is synthesized in the liver, it is esterified into PLs, assembled into lipoproteins, and secreted into the blood where it is hydrolyzed again by endothelial lipases and taken up by tissues [7, 15].

### DHA entrance into the brain by crossing the blood-brain barrier

The central nervous system (CNS) has the second greatest amount of lipids of the body and ~35 % are polyunsaturated fatty acids (PUFA), being DHA and arachidonic acid (AA), the two major PUFA [16, 17]. DHA is the most predominant fatty acid (FA) found at the sn-2 position in PLs on neuronal and synaptic membranes [18].

Most of the DHA that constitutes the brain before birth and during breast feeding is supplied from the mother [19]. After weaning, DHA is supplied mainly by the liver, where it can be synthesized from its precursors, αLNA, and EPA [19]. DHA is incorporated into the brain from the blood [20]. It has been shown that unesterified DHA can diffuse through the blood-brain barrier (BBB) and readily enter the brain [21]. However, DHA in the blood is bound to serum albumin (SA), either as an unesterified FA or esterified as DHA-lysophosphatidylcholine (DHA-LPC) [22]. After being released from SA, DHA is able to cross the BBB, mainly in the form of DHA-LPC [22]. Although passive diffusion of DHA has been shown, PUFA 18 and 20 carbons long can enter the brain through FA transporter proteins (FATP) and FA binding proteins (FABP), as shown in human brain microvessel endothelial cells [23]. Specially, FATP4, FABP5, and fatty acid translocase/CD36 mediate PUFA transport [23]. In the hippocampus of primates, FABP5 is expressed in neurons while FABP7 is found in astrocytes [24], suggesting a possible mechanism for DHA transport. Also, a previously identified orphan sodium-dependent LPC symporter, *Mfsd2a*, has been implicated in the transport of DHA-LPC but not free DHA across BBB microvessels [25]. In this sense, it has been shown that DHA-LPC esterified at the sn-2 position is captured by the brain more efficiently than free DHA in rats [26, 27].

In the rat brain, there is evidence that astrocytes are capable of synthesizing DHA continuously [28, 29] and that endothelial cells from microvasculature, astrocytes, and neurons synthesize DHA and cooperate for DHA accretion in the brain [30]. Interestingly, DHA accretion decreases slightly after the administration of a high concentration of DHA in the mouse brain [21] and in cultured astrocytes [29], suggesting that some mechanisms regulate DHA entrance and synthesis when DHA levels increase. However, in these experiments, DHA levels were not affected importantly [21, 29]. Finally, it has been suggested that DHA conservation mechanisms might exist in the brain, as has been shown in retinal pigment epithelium [31], suggesting that DHA entrance and production in the brain are sensed and regulated.

### DHA and DHA derivatives are involved in neuroprotection through peroxisome proliferator-activated receptors

DHA in the brain of humans and other vertebrates participates in normal growth, development, and function [32], acting as a neurotrophic factor [33] and modulating synaptic activity [34]. Interestingly, DHA-oxygenated derivatives are known to be produced during strokes in the murine brain [35] and to prevent leukocyte infiltration in ischemic murine models [36], thus modulating inflammation in the brain.

DHA derivatives are produced from the DHA contained in the acyl chains of PLs of cellular membranes. In the cellular membrane, DHA is cut by the action of iPLA_2_ [37, 38]. Subsequently, lipoxygenases (LOX) [36, 37, 39, 40] and/or cyclooxygenase 2 (COX2, induced by stress stimuli) [35, 41] produce di- and trihydroxylated DHA derivatives, called resolvins or protectins (docosanoids) [36, 37, 39–41]. The first DHA derivatives described were called resolvins, due to their anti-inflammatory activity in murine brain exudates [41]. In particular, the 7S,8R,17S-trihydroxy-docosa-4Z,9E,11E,13Z,15E,19Z-hexaenoic acid called resolvin D1 (RvD1) has been shown to participate in resolution of inflammation [41, 42]. Another studied DHA derivative that acts as a neuro-protector is the 10R,17S-dihydroxy-docosa-4Z,7Z,11E,13E,15Z,19Z-hexaenoic acid, called neuroprotectin or protectin D1 (NPD1) [36, 40], shown to halt the inflammatory response by decreasing the number of cytotoxic T cells and their migration, as well as the production of pro-inflammatory mediators [36].

**Fig. 1:**
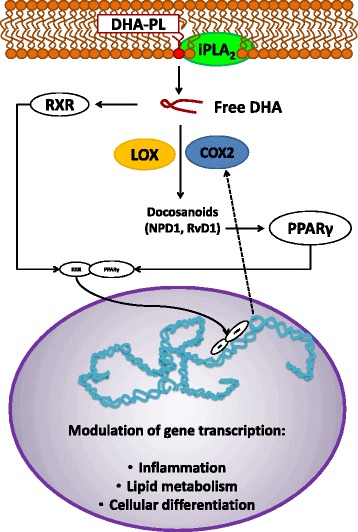
DHA modulation of gene transcription through the action of RXR/PPARγ transcription factors heterodimers. DHA is cut from PLs in cellular membranes by the action of iPLA_2_. DHA is oxygenated by the action of LOX, or alternatively by the action of COX2. The products of DHA oxygenation are docosanoids, such as NPD1 and RvD1. DHA is a ligand for RXR and NPD1 for PPARγ. Activation of RXR and PPARγ leads to the formation of RXR/PPARγ, which binds to a PPAR response element, in the promoter region of target genes [38], thus modulating inflammation, lipid metabolism, and cellular differentiation. *DHA* docosahexaenoic acid, *PL* phospholipids, *iPLA*_*2*_ independent phospholipase A_2_, *LOX* lipoxygenases, *COX2* cyclooxygenase 2, *NPD1* neuroprotectin D1, *RvD1* resolvin D1, *RXR* retinoid X receptor, *PPARγ* peroxisome proliferator-activated receptors gamma, *RXR/PPARγ* heterodimers of nuclear transcription factors RXR and PPARγ. Activation is indicated by *black arrows* and modulation by the *black gaped arrow*

DHA is an endogenous ligand for retinoid X receptors (RXR), which form heterodimers with peroxisome proliferator-activated receptors (PPARs) to produce nuclear transcription factors RXR/PPAR, and these can modulate gene expression in different cell types [38, 43]. Additionally, NPD1, but not DHA, activates the PPAR-γ isoform (PPARγ) in human neuronal and glial cells [37]. DHA-oxygenated synthetic derivatives are also potent activators of PPARγ [44], and PPARs lead in the murine model to the control of differentiation or neurons and astrocytes [45, 46]. Furthermore, PPARs modulate COX2 activity in the murine brain [47], suggesting a feedback loop in DHA signaling because COX2 participates in DHA oxygenation [35, 41]. Thus, DHA and DHA derivatives can exert gene modulation through RXR/PPARγ activation, dimerization, and translocation to the nucleus (Fig. 1).

### DHA modulates the inflammatory response in AD

Alzheimer’s disease (AD) is a neurodegenerative disease that leads to dementia. It is characterized histologically by amyloid deposits, constituted by aggregates of amyloid β peptide (Aβ) and neurofibrillary tangles, conformed by aggregates of microtubule-associated tau protein and cell loss [48, 49]. Aβ is the cleavage product of amyloid β protein precursor (APP) by the β site cleavage enzyme 1 (BACE1) and the γ-secretase complex. Overproduction or inefficient removal of Aβ is thought to trigger early damage in AD [48, 49]. Aβ can be aggregated into oligomeric soluble species, fibrils, and finally amyloid plaques or deposits, leading to glia activation and the induction of an inflammatory response [50, 51].

### Participation of glial cells in AD

Glial cells, astrocytes, and microglia, the main supportive cells of neurons, are encountered near Aβ plaques [52, 53]. Nevertheless, the role of glial cells in the pathology of AD is not clear. Whereas inactivated or dysfunctional glial cells increase the amyloid burden and AD pathology, activation of glial cell leads to the production of cytotoxic molecules like nitric oxide (NO), thus contributing to inflammatory damage [50–53].

### Astrocytes

**Fig. 2:**
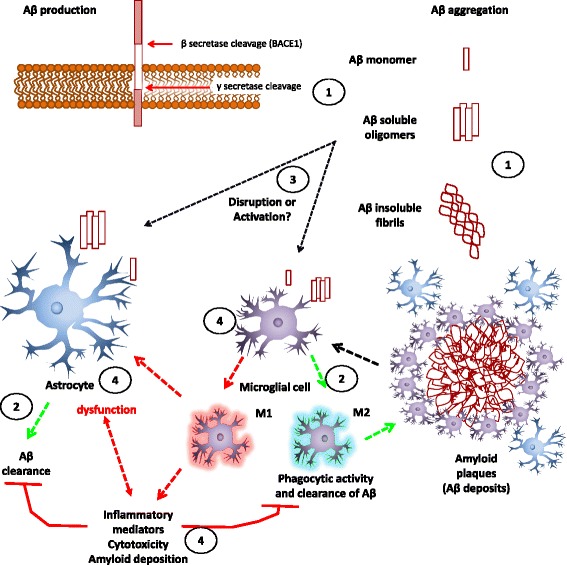
Participation of glial cells in AD. *1* Aβ is secreted by the action of BACE1 and γ-secretase, thus forming aggregates of Aβ (oligomers, insoluble fibrillary aggregates and plaques), *2* microglia (M2- like phenotype) and astrocytes capture and degrade Aβ peptides, *3* Aβ aggregates can activate or harm microglia and astrocytes, promoting the production of pro-inflammatory cytokines and mediators, *4* dysfunction of microglia and astrocytes allows Aβ deposition, increasing cellular damage and inflammation, *BACE1* β site cleavage enzyme 1, *Aβ* amyloid β peptide, *M1* pro-inflammatory microglia, *M2* anti-inflammatory microglia, *green arrows* anti-inflammatory and clearing action, *red arrows* pro-inflammatory actions, *black arrows* interaction

Astrocytes are part of the BBB, providing protection to neurons by secreting neurotrophic factors. They nurture neurons and release neurotransmitters that sustain neuronal synaptic transmission [51–54]. In AD, astrocytes are associated with the amyloid pathology and an inflammatory environment, and when activated, they acquire a greater size and express higher levels of glial acid fibrillary protein (GFAP) [52]. In addition, astrocytes can become dysfunctional by Aβ, especially by toxic oligomeric species that lead to calcium dyshomeostasis and finally disrupt astrocytic support of neuronal synaptic function (reviewed in [55]). Astrocytes are structural components of the BBB, and cerebral-vascular deficiencies increase the influx and buildup of Aβ in the brain. This further contributes to the sustained activation of glia (microglia and astrocytes) and to the secretion of two pro-inflammatory molecules, tumor necrosis factor-α (TNF-α), and interleukin-1β (IL-1β), which in turn disrupts glutamatergic transmission [55]. In addition, astrocytes express proteins that are involved in the clearance of Aβ. The failure of neprilysin, insulin-degrading enzyme, and matrix metalloproteinase 9 (proteolytic enzymes) to degrade Aβ promotes the buildup of Aβ in the brain parenchyma, the activation of glial cells, and even a greater Aβ secretion by astrocytes (reviewed in [56]). Moreover, apolipoprotein E (APOE) is needed for Aβ clearance by astrocytes, and bearing APOE ε4 allele decreases Aβ clearance and increases Aβ deposition [57]. The sustained activation of astrocytes increases the production of cytotoxic mediators, such as NO, complement protein, and reactive oxygen species (ROS) [57]. For example, increased production of the complement factor 3 protein in the presence of Aβ and the activation of the nuclear transcription factor kappa-light-chain-enhancer of activated B cells (NF-κB) has been suggested to impair the synaptic function of neurons and the behavior of mice in a model of AD [58]. However, these results are controversial, and the rational of the experimental methods has been debated [59]. Finally, activated astrocytes lead to the release of chemokines such the C-X-C motif ligand 10 (CXCL10), which in turn attracts microglia to Aβ sites through the C-X-C chemokine receptor 3 (CXCR3) [52, 53] (Fig. 2).

### Microglia

Microglial cells are the immune-competent cells of the CNS and the primary phagocytic cell responding to Aβ. When isolated and exposed to Aβ, microglia from AD brain tissue shows elevated expression of interleukin 1 (IL-1), TNF-α, interleukin 6 (IL-6), and interleukin 12 (IL-12). This strongly suggests that either Aβ or amyloid plaques can initiate the activation of microglia and the subsequent release pro-inflammatory molecules in the AD brain [52, 53]. Depending on their activation state, microglia may contribute to the toxic milieu in AD. Like macrophages, microglia exhibit the pro-inflammatory (M1) phenotype, characterized by the expression of cytotoxic genes TNF-α, IL-1, IL-6, IL-12, and interleukin 18 (IL-18), as well as impaired phagocytic capacity [57]. The anti-inflammatory (M2) phenotype, on the other hand, is characterized by expression of anti-inflammatory molecules interleukin 4 (IL-4), interleukin 10 (IL-10), interleukin 13 (IL-13), and transforming growth factor β (TGF-β), as well as by increased phagocytic capacity without production of the toxic NO [57]. However, these two states of activation are assumed to be the extremes of a variety of activation states, and all can contribute differently to AD progression [57]. In addition, aging can enhance microglia’s sensitivity, exacerbating the inflammatory response [53, 57], thus favoring the M1 phenotype by decreasing the phagocytic ability of microglia. Nevertheless, microglia are activated and migrate to amyloid deposits early in the pathogenesis of a mouse model of AD [60]. In old AD patients, microglia from the hippocampus is the prominent proliferating cell population surrounding amyloid deposits [61]. Also, an increased number of activated microglia are detected in the dentate gyrus of hippocampus in the triple transgenic mouse model of AD (3xTg-AD) before overt plaque deposition [62], suggesting that microglia respond early in the pathology of AD and become more active than astrocytes. It is unclear whether this contributes to the pathology or is a mechanism to contain Aβ damage. Additionally, microglia are more effective than astrocytes for phagocyting fibrillar Aβ, except when the latter are associated to APOE, apolipoprotein J (APOJ), α1-antichymotrypsin, or serum amyloid P-complement C1q protein, which are present in amyloid plaques. Furthermore, the same proteins impair phagocytosis of oligomeric Aβ by astrocytes [63]. Thus, the data suggests that both microglia and astrocytes may have differential roles in inflammation during the pathology of AD and that their dysfunction may contribute to the damage in AD (Fig. 2).

### DHA and its derivatives as modulators of glial cell activity

A large amount of evidence, in cellular and animal models under neurotoxic stimuli, has suggested that DHA can prevent inflammation by modulating glial cell activity [64–67]. In vitro, DHA diminishes the activation of microglia, the production of pro-inflammatory cytokines such as TNF-α, IL-1β, and IL-6, and the production of the chemokines C-C motif ligand 2 (CCL2), C-C motif ligand 3 (CCL3), and CXCL10 [67]. In microglia derived from mice, DHA decreases the release of NO that is induced by lipopolysaccharide (LPS) or interferon γ (IFN-γ) and myelin [68]. DHA reduces production of the pro-inflammatory cytokines and NO induced through toll-like receptors type 3 and 4 (TLR3 and TLR4) [69]. This is explained in part by DHA incorporation in the cellular membrane phospholipids that impairs the presentation of antigens, including LPS by TLR4 and its associated receptor CD14 molecule (CD14), thus preventing NF-κB activation and synthesis of IL-1β and TNF-α [70]. On the other hand, DHA inhibits p38 mitogen-activated protein kinase (p38 MAPK) phosphorylation [67, 71], thus inhibiting the expression of inflammatory molecules, and promotes the activation of PPARγ [71], which modulates lipid and glucose metabolism. In microglia exposed to LPS, DHA inhibits the pro-inflammatory characteristics of microglia, including enlarged lipid bodies (lipid droplets, composed of neutral lipids, mainly phospholipids, sterols, triacylglycerols, sterol esters, and proteins) [72], involved in the production of inflammatory mediators [73] and NO. Hence, this mechanism of DHA lessens dendritic damage associated with inflammation in hippocampal slices of mice [74].

In vitro and in vivo, microglia pro-inflammatory activity is associated with an ameboid-like phenotype and the expression of pro-inflammatory cytokines and chemokines related to the M1 macrophage phenotype. DHA treatment reduces ameboid morphology in Müller’s glia (microglia) in the retina [75] and leads to a phenotype with extended branches and the expression of molecules of the M2 macrophage phenotype, related to termination of inflammation [68]. Nevertheless, it is also possible that DHA inhibits the synthesis of pro-inflammatory mediators without inducing a change in phenotype of microglia, despite prompting the inhibition of p38 MAPK phosphorylation and the activation of PPARγ, related to anti-inflammatory action [71]. Furthermore, DHA anti-inflammatory effects are accompanied by an increase in the phagocytic activity of microglial cells [68]. In a human microglial cell line (CHME3), DHA stimulates Aβ phagocytosis and promotes an anti-inflammatory profile [76]. Apart from its anti-inflammatory action, DHA promotes antioxidant activity in BV-2 microglia, by up-regulation of heme oxygenase-1 (HO-1) and protein kinase B (AKT) activation [77]. In addition, increased total glutathione levels have been found in microglia cells under DHA administration [69], supporting DHA role in antioxidant activity in these cells, which could protect them against the oxidative damage associated with AD.

Supplementation of DHA in rodents and humans demonstrates anti-inflammatory action and tissue protection in microglia. DHA enhanced photoreceptor survival and converted activated microglia into a quiescent phenotype in retinal sections of retinoschisin (Rs1h)-deficient mice [78], resembling that of phagocytic non-inflammatory microglia. After induction of cerebral ischemia, DHA reduces central macrophage/microglia activation, leukocyte infiltration, peripheral leukocyte activation, and expression of TNF-α, IL-1β, IL-6, monocyte chemotactic protein-1 (MCP-1), and the CCL2 receptor (CCR2). Also, post-stroke oxidative stress decreases with DHA supplementation, as demonstrated by low c-Jun N-terminal kinase (JNK) phosphorylation, as well as activation of c-Jun phosphorylation and activating protein-1 (AP-1), and an elevated expression of NF-E2-related factor-2 (Nrf2) and HO-1 [79]. In rats with injured sciatic nerve, DHA treatment significantly reduces neurogenic pain and neuronal damage by reducing allograft inflammatory factor 1 (AIF, also known as induction of brown adipocytes, iba-1), positive satellite cells (macrophages or microglia) and expression of the pro-apoptotic p53 protein in the dorsal root ganglia (DRG) [66]. The review of Hjorth and Freund-Levi deals with DHA and EPA action on microglial cells [80].

DHA modulation of astrocytes also demonstrates fine tuning of neuronal activity through inhibition of pro-inflammatory mediators and an important regulation of astrocytic activity. Astrocytes cultured from the rat brain and pre-incubated with DHA prevent the cytotoxic effects of excessive unconjugated bilirubin (UCB) and increase the activity of superoxide dismutase (SOD), catalase (CAT), and glutathione peroxidase (GPx), therefore contributing to the antioxidant defense [81]. DHA-treated astrocytes also decrease the production of TNF-α and IL-6 while attenuating the phosphorylation of both p38 and p42/44 MAKP, suggesting modulation of TLR4 [82]. Following in vitro ischemia, DHA prevents calcium dyshomeostasis and endoplasmic reticulum stress (ERS) in astrocytes by acting on inositol 1,4,5-triphosphate receptors, attenuating the phosphorylation of eukaryotic initiation factor 2α (EIF2α) and activating transcription factor-4 (ATF-4) following in vitro ischemia [83]. Importantly, DHA modulates glutamate transmission by regulating glutamate transport in astroglia [84] and thus can potentially alleviate the dysregulation of calcium homeostasis and glutamatergic transporters associated with AD [55]. The importance of DHA levels in glutamate recycling by astrocytes has been explored by Latour and collaborators, showing that low levels of DHA in rats are associated with increased astroglial expression of GFAP and decreased uptake of glutamate by astrocytes [85].

Like DHA, the docosanoid NPD1 promotes a ramified, non-inflammatory microglial phenotype and attenuation of choroidal neo-vascularization when administered topically in the eye of mice [86]. The NPD1 isomer 10S,17S-dihydroxy-docosa-4Z,7Z,11E,13Z,15E,19Z-hexaenoic acid (DiHDoHE) has similar effects as DHA on calcium dyshomeostasis and ERS in astrocytes [83].

RvD1 inhibits LPS and IFN-γ-induced TNF-α release in astrocyte cultures by inhibition of the extracellular-regulated mitogen-activated protein kinase (ERK) [87]. In peripheral blood mononuclear cells (PBMC) from AD patients, incubated with Aβ, RvD1 promotes the phagocytosis of Aβ in vitro, inhibits apoptosis through the chemokine receptor G protein-coupled receptor 32 (GPR32), and promotes anti-inflammatory profiles by up-regulation of the interleukin 1 receptor antagonist (IL1RN), the integrin B 2 protein (ITGB2), and NF-κB expression, along with the down-regulation of pro-inflammatory cytokines, such as IL-1 and IL-6 [88]. In summary, DHA and its derivatives down-regulate the expression of pro-inflammatory mediators related to cytotoxic cell damage, while up-regulating the expression of anti-inflammatory mediators. This profile prevents the recruitment of resident or incoming immune cells and promotes phagocytic and antioxidant activity. The effects of DHA and its derivatives are summarized in Table 1.

**Table 1:** Anti-inflammatory effect of DHA or derivatives on glial cells

DHA or derivative	Cell type	Effect	Model	Reference
DHA	Astrocytes	−TNF-α and IL-6	In vitro primary cell culture from rat brain	[83–86]
		+Antioxidant enzymes		
		−Calcium dyshomeostasis		
		+Glutamate uptake		
		−ERK		
		−MAPK		
RvD1	Astrocytes	−TNF-α and ERK	In vivo and in vitro primary cell culture from rat and human cell line	[89]
DHA	Glia	−TNF-α	In vitro primary cell culture from rat brain	[79]
		−IL-1β		
		−IL-6		
		−MCP-1 and CCR2		
DHA	Microglia	+Phagocytic activity	In vitro primary cell culture from rat and mice brain and cell lines	[67–71, 77]
		−p38 MAPK		
		−TNF-α		
		−IL-1β		
		−IL-6		
		CCL2, CCL3, and CXCL10		
		−NO		
		+Glutathione		
		−NOS		
		−COX2		
		−TLR4 and CD14		
		−NFκB		
DiHDoHE	Microglia	−Ameboid morphology	Choroidal neovascularization in rats	[88]
DHA	Macrophages/Microglia	−Infiltration	Neurologic pain in rats	[66, 79]
		−CD45^high^/CD11b^high^		
		−Pro-inflammatory cytokines	Cerebral ischemia in rats	
		+Anti-oxidative pathway		
DHA	Müller glia	−Ameboid morphology	Retina of CLN6^NCLF^ mice	[75]

*+* increase or activation,*−* decrease or inhibition, *CCL2* C-C motif ligand 2, *CCL3* C-C motif ligand 3, *CCR2* CCL2 receptor, *CD14* receptor cluster of differentiation 14, *COX2* cyclooxygenase 2, *DHA* docosahexaenoic acid, *DiHDoHE* 10S,17S-dihydroxy-docosa-4Z,7Z,11E,13Z,15E,19Z-hexaenoic acid, *ERK* extracellular-regulated mitogen-activated protein (MAP) kinase, *IL-1β* interleukin 1β, *IL-6* interleukin 6, *MCP-1* monocyte chemotactic protein-1, *NF-κB* nuclear factor kappa-light-chain-enhancer of activated B cells, *NO* nitric oxide, *p38 MAPK* p38 mitogen-activated protein kinase, *RvD1* resolvin D1, *TLR4* toll-like receptor type 4, *TNF-α* tumor necrosis factor-α, *CD45* ^*high*^ */CD11b* ^*high*^ activated macrophage/microglia [79], *CD45* protein tyrosine phosphatase, receptor type C, *CD11b* integrin subunit alpha M (ITGAM), *CLN6* ^*NCLF*^ *mouse* a mouse with natural occurring neuronal ceroid lipofuscinoses (*NCLF*) and alterations in the ceroid-lipofuscinosis, neuronal 6 (*CLN6*) gene

### Activation of PPARγ is involved in the modulation of glia by docosanoids

**Fig. 3:**
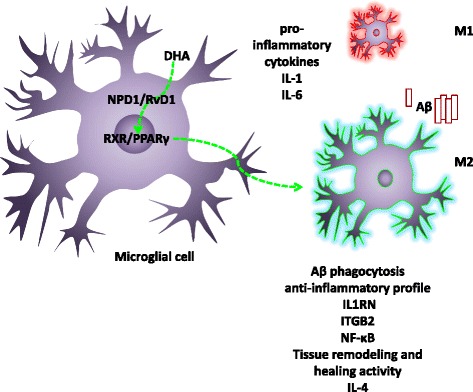
DHA and docosanoids modulate the activation of microglia. DHA and/or docosanoids activate RXR/PPARγ heterodimers that promote transcription of anti-inflammatory cytokines and acquisition of M2 anti-inflammatory profile [74–78]. *IL-1* interleukin 1, *IL1RN* interleukin 1 receptor antagonist, *ITGB2* integrin B 2 protein, *IL-4* interleukin 4, *IL-6* interleukin 6, *NF-κB* nuclear factor kappa-light-chain-enhancer of activated B cells, *Aβ* amyloid β peptide, *DHA* docosahexaenoic acid, *NPD1* neuroprotectin D1, *RvD1* resolvin D1, *RXR/PPARγ* heterodimers of nuclear transcription factors RXR and PPARγ, *M1* pro-inflammatory microglia, *M2* anti-inflammatory microglia, *green arrows* anti-inflammatory and clearance action

Although there is no clear evidence that DHA is a direct ligand of PPARγ, in silico and in vitro analysis suggests that carboxyl group of DHA can form hydrogen bonds with four of the five amino acids in the ligand-binding pocket of the PPARγ molecule [44]. Furthermore, synthetic-oxygenated derivatives of DHA, such as 5E-4-hydroxydocosahexaenoic acid (4-HDHA) and 5E-4-oxodocosahexaenoic acid (4-oxoDHA), bind and activate PPARγ even more potent than pioglitazone [44]. RvD1 promotes the expression of markers in microglia associated with tissue remodeling and healing activity. In this sense, RvD1 enhances IL-4 as well as the activation of the signal transducer and activator of transcription 6 (STAT6) and the PPARγ transcriptional factors [64]. Additionally, NPD1 activates PPARγ in human neurons and glial cell co-cultures. Despite the important decrease in Aβ secretion caused by NPD1, its inflammatory response has not been evaluated [37]. Moreover, activation of heterodimeric PPARγ/RXRα results in increased phagocytosis of Aβ by microglia [89]. In this sense, PPAR-α isoform agonists diminish the inflammatory response of microglia [65], thus making PPARs or PPARγ potential modulation targets of docosanoids. Nevertheless, it is necessary to establish methods for delivering a particular docosanoid in humans (Fig. 3).

### DHA and its derivatives modulate the cellular lipidic environment and amyloid production

DHA modulation of the lipid composition and organization of cellular membranes has been greatly studied. Particularly, it is well known that cholesterol levels are modulated by DHA [90–92]. DHA acyl chains incorporate directly into the lipid raft micro-domains and exclude cholesterol, thus changing protein organization, clustering activity, and signaling [33, 91, 92]. Alternatively, DHA halts cholesterol synthesis and reduces the γ-secretase contents in lipid rafts and activity of BACE1 [93]. Thus, the effects of DHA on the lipidic composition of cellular membranes are of particular interest, because in the brains of AD patients, highly ordered membrane lipid rafts have been found with increased levels of cholesterol, which in turn contain increased levels of BACE1 that suggests increased amyloidogenic processing of APP [94].

In the same sense, DHA administration to transgenic APPswe/PS1dE9 mice, which overproduce Aβ, has proven to lessen Aβ production while decreasing the ratio of omega-6/omega-3 [95]. Additionally, DHA stimulates the production of the soluble amyloid precursor protein α (sAPPα), the alternative non-toxic product of the APP cleavage, and decreases Aβ secretion [96]. The DHA derivative 2-hydroxydocosahexaenoic acid (2OHDHA) shows similar effects on amyloidosis [97], and NPD1 decreases Aβ secretion and protects a primary co-culture of human neurons and glia by increasing sAPPα, which further increases NPD1 production [98]. In addition, NPD1 down-regulates BACE1 expression and activity through the activation of PPARγ [37], a known target of docosanoids [44]. Thus, apart from altering the lipidic profile of cellular membranes, DHA can also modulate the enzymes involved in APP processing and Aβ production.

DHA prevents Aβ toxicity by directly interacting with Aβ monomers or oligomers, thus preventing fibril formation, and lipid peroxidation, as well as increasing the viability of neuronal cells in vitro [99]. Additionally, 2OHDHA acid, a DHA derivative, promotes Aβ monomer insertion into the cellular membrane (rather than the oligomeric or fibrillar Aβ species), thus suggesting the prevention of Aβ oligomerization and derived toxicity [97]. Furthermore, it has been observed that Aβ short species (25–35) form annular structures that solubilize artificial lipid membranes. Increasing DHA levels in the cellular membrane prevents its solubilization and promotes the disruption of Aβ annular structures [100].

### DHA treatment in AD: from research to clinical evidence

Since cognitive deficits are the clinical hallmark of AD, any prospective treatment has to be able to ameliorate cognitive deficiencies in animal models and finally in humans. In this regard, DHA has proven to be protective against cognitive deficits in transgenic and non-transgenic murine models of AD by restoring dendritic spine molecular functionality [101, 102], reducing Aβ load, helping against Aβ toxicity [103–106], decreasing tau pathology [107], and increasing cerebral blood volume [108]. Furthermore, administration of 2OHDHA in the 5XFAD transgenic mouse model of AD improved memory and restored cell proliferation in the dentate gyrus without changing the content of Aβ plaques, suggesting that cell proliferation is a major component of memory recovery in mice [109].

In patients with AD (from the OmegaAD study), DHA supplementation increased DHA levels in cerebral spinal fluid and directly correlated with soluble interleukin-1 receptor type II, an inhibitor of IL-1, and the modulation of genes involved in the inflammatory response [110, 111]. This suggests an anti-inflammatory effect of DHA in humans. However, DHA has proven to be ineffective in improving cognition in AD patients with mild to moderate dementia, although some data demonstrate effectiveness in treating patients with mild cognitive impairment (MCI, a prodrome to AD) and participants with memory complaints [112].

What could explain DHA ineffectiveness, despite all the data pointing to DHA benefits? APOE ε4 is a risk factor for developing AD and has been shown to further reduce the effectiveness of DHA treatment. A clinical study conducted in conjunction with the Alzheimer’s Disease Cooperative Study (ADCS) showed that 2 g/day of DHA had no effect on the cognitive state or brain atrophy of AD patients. Nevertheless, AD patients negative for APOE ε4 showed mild though not significant effectiveness of this treatment [113]. In this sense, influence of the APOE ε4 allele on DHA metabolism has been studied in healthy individuals, showing lower levels of DHA in plasma and a lower half-life of DHA in the body [114]. Also, when comparing mice carrying the APOE ε4 allele with those having the APOE ε2 allele, the former show lower brain uptake of DHA [115]. APOE proteins are found on the surface of chylomicron particles after lipid consumption and serve as ligands for low-density lipoprotein receptors, thus regulating the transport of FA. The APOE genotype (ε2, ε3, or ε4) modifies the rate of clearance of ω-3 FA, and APOE ε4 accelerates the clearance of ω-3 FA, increasing β-oxidation of DHA [116] and potentially disrupting lipid metabolism [117]. However, the authors suggest that results on DHA metabolism are inconclusive and that the analysis need to be improved [116, 117]. Interestingly, astrocytes from an AD mice model treated with liver X receptor (LXR) agonists express APOE. Moreover, conditioned medium of primary astrocytes from AD mice increased Aβ phagocytosis that relies on APOE and LXR expression [118]. DHA in combination with the LXR agonist bexarotene increases APOE expression, which in turn increases phagocytosis and reduces inflammatory mediators in astrocytes [119]. Interestingly, bexarotene also induces APOE ε4 lipidation, which increases the generation of APOE ε4–Aβ complexes and reduces Aβ pathology as well as synaptic damage in a mouse model expressing human APOE [120], suggesting a potential role of bexarotene for treating AD pathology and defective APOE ε4; however, bexarotene safety must be guaranteed [120].

There is evidence of DHA deficiency in the plasma from patients diagnosed with AD [121–124]. However, there is controversy concerning DHA levels between AD patients and cognitive normal subjects [125]. Epidemiological studies associate scarce DHA consumption with an increased risk of AD [113]. In a similar sense, mouse models evidence reduced DHA during the aggravation AD pathology [126]. DHA is the major ω-3 PUFA in synaptic membranes. A decreased level of DHA in hippocampus synapses is characterized by altered synaptic transmission and glutamate release by neurons [127], deficient uptake of glutamate by astrocytes, and altered extracellular levels of glutamate [85]. However, restoration of DHA levels by ω-3 supplementation improves altered synaptic transmission and glutamate release [127].

In light of these findings in humans, DHA metabolism in AD patients with APOE ε4 may be especially disrupted, and the effectiveness of DHA treatment might be compromised. This suggests a possible subgroup for DHA administration: those at risk for developing AD with no overt cognitive deterioration and without bearing the APOE ε4 allele.

In this respect, effectiveness of fish oil or DHA-enriched fish oil has been proved in recent studies. DHA-enriched fish oil supplementation in MCI has been observed in a double-blind Malayan study. Subjects over 60 years old received DHA-enriched fish oil during a year, a treatment that improved memory and attention [128]. Other supplements containing ω-3, including DHA, have also shown stabilization of the cognitive state in MCI, but as found in previous studies, neither improvement nor stabilization was observed in AD patients [129]. Finally, a recent published retrospective study evaluated the effect of fish oil consumption on cognition and brain volume in normal individuals as well as MCI and AD patients. By using a generalized estimating equation (GEE) model to analyze incomplete longitudinal data on the Alzheimer’s Disease Neuroimaging Initiative (ADNI) from inception to August 2010, it was found that in all cases, fish oil consumption was associated with increased brain volume and a better cognitive performance, except in APOEε4 carriers as observed in former studies [130].

However, the efficacy of DHA alone has to be reevaluated as it may be more effective in combination with other nutrients including vitamin B [131], or compounds such as bexarotene [119]. Alternative treatments may include the activation of PPARs with agonists or DHA derivatives [132]. Overall, a reevaluation of ω-3 and especially DHA may provide valuable information about DHA effectiveness on MCI.

## Conclusions

DHA is a natural compound that can easily be obtained from animal sources, especially cold-water fish. DHA can easily cross the BBB, especially in the form of the DHA-LPC, and therefore, it is suitable as a therapeutic agent for neurological disorders. DHA, free or bound in to PLs, is incorporated into cellular membranes, where it is released and transformed into docosanoids (oxygenated derivatives) to exert its function within the cells via RXR and PPARγ. Although DHA has been associated with protection, based on the modification of cellular membrane fluidity, increasing data suggest that DHA’s action can be attributable to a signaling cascade in which docosanoids exert their action by regulating gene expression of anti-inflammatory and other protective pathways. The neurodegenerative disease of Alzheimer has a complex etiology in which the deposition of Aβ plays an important role. Inflammation is an early event in AD that contributes to increased neuronal damage, especially due to the dysfunction of glial cells. This dysfunction leads to the lack of clearance of Aβ, which further increases the over-activation of glial cells. Overall, a harmful environment is created, withdrawing support to neurons and favoring plaque formation.

In animal models and in vitro, DHA and its derivatives have proven to regulate gene expression of inflammatory mediators, as well as enzymes involved in lipid metabolism and Aβ processing. Activation of PPARγ has been shown to mediate some of the effects promoted by DHA and its derivatives in neurons and glial cells. Therefore, based on the current evidence, DHA or its derivatives can help to prevent or retard inflammatory aspects of the pathology of AD. This aspect of the disease has been considered to be an important player in the determination of the outcome of AD. On the other hand, patients carrying the APOE ε4 allele show disrupted metabolism of DHA. Further evaluation of this trait is needed. New longitudinal studies considering early symptoms of cognitive deterioration associated to AD, including MCI, DHA metabolism in APOE ε4 participants, and inflammatory status, might help to conclude whether people at risk of developing AD can potentially be treated with DHA.

## Abbreviations

2OHDHA: 2-hydroxydocosahexaenoic acid
3xTg-AD: triple transgenic mouse model of AD
4-HDHA: 5E-4-hydroxydocosahexaenoic acid
4-oxoDHA: 5E-4-oxodocosahexaenoic acid
AA: arachidonic acid
AD: Alzheimer’s disease
ADCS: Alzheimer’s Disease Cooperative Study
ADNI: Alzheimer’s Disease Neuroimaging Initiative
AIF: allograft inflammatory factor 1, also known as induction of brown adipocytes, iba-1
AP-1: activating protein-1
APOE: apolipoprotein E
APOJ: apolipoprotein J
APP: amyloid β precursor protein
ATF-4: activating transcription factor-4
Aβ: amyloid β peptide
BACE1: β site cleavage enzyme 1
BBB: blood-brain barrier
CAT: catalase
CCL2: C-C motif ligand 2
CCL3: C-C motif ligand 3
CCR2: CCL2 receptor
CD11b: integrin subunit alpha M (ITGAM)
CD14: CD14 molecule
CD45: protein tyrosine phosphatase, receptor type C
CLN6^NCLF^mouse: a mouse with natural occurring neuronal ceroid lipofuscinoses (NCLF) and alterations in the ceroid-lipofuscinosis, neuronal 6 (CLN6) gene
CNS: central nervous system
COX2: cyclooxygenase 2
CXCL10: C-X-C motif ligand 10
CXCR3: C-X-C chemokine receptor 3
DHA: docosahexaenoic acid
DiHDoHE: 10S,17S-dihydroxy-docosa-4Z,7Z,11E,13Z,15E,19Z-hexaenoic acid
DRG: dorsal root ganglia
EIF2α: eukaryotic initiation factor 2α
EPA: eicosapentaenoic acid
ERK: extracellular-regulated mitogen-activated protein kinase
ERS: endoplasmic reticulum stress
FA: fatty acid(s)
FABP: FA binding protein(s)
FATP: FA transporter protein(s)
GFAP: glial acid fibrillary protein
GPR32: G protein-coupled receptor 32
GPx: glutathione peroxidase
HO-1: heme oxygenase-1
IFN-γ: interferon γ
IL-1: interleukin 1
IL-10: interleukin 10
IL-12: interleukin 12
IL-13: interleukin 13
IL-18: interleukin 18
IL1RN: interleukin 1 receptor antagonist
IL-1β: interleukin 1β
IL-4: interleukin 4
IL-6: interleukin 6
iPLA_2_: independent phospholipase A_2_
ITGB2: integrin B 2 protein
JNK: c-Jun N-terminal kinase
AKT: protein kinase B
LA: linoleic acid
LCPUFA: long-chain polyunsaturated fatty acid
LOX: lipoxygenases
LPC: lysophosphatidylcholine
LPS: lipopolysaccharide
LXR: liver X receptor
M1: pro-inflammatory microglia
M2: anti-inflammatory microglia
MAPK: mitogen activated protein kinase
MCI: mild cognitive impairment
MCP-1: monocyte chemotactic protein-1
NF-κB: nuclear factor kappa-light-chain-enhancer of activated B cells
NO: nitric oxide
NPD1: neuroprotectin D1 or protectin D1
Nrf2: NF-E2-related factor-2
p38 MAPK: p38 mitogen-activated protein kinase
PBMC: peripheral blood mononuclear cells
PLs: phospholipids
PPARs: peroxisome-proliferator activated receptors
PPARγ: PPAR-γ isoform
PUFA: polyunsaturated fatty acid(s)
ROS: reactive oxygen species
RvD1: resolvin D1
RXR: retinoid X receptors
SA: serum albumin
sAPPα: soluble amyloid precursor protein α
SOD: superoxide dismutase
STAT6: signal transducer and active tor of transcription 6
TAG: triacylglycerol
TGF-β: transforming growth factor β
TLR3: toll-like receptor type 3
TLR4: toll-like receptor type 4
TNF-α: tumor necrosis factor-α
UCB: unconjugated bilirubin
αLNA: α-linolenic acid
Δ6D: Δ6-desaturase
ω-3: omega-3
